# Systematic and Bibliometric Analysis of Magnetite Nanoparticles and Their Applications in (Biomedical) Research

**DOI:** 10.1002/gch2.202200009

**Published:** 2022-09-14

**Authors:** Charlotte L. Fleming, Mojtaba Golzan, Cindy Gunawan, Kristine C. McGrath

**Affiliations:** ^1^ School of Life Sciences Faculty of Science University of Technology Sydney Sydney NSW 2008 Australia; ^2^ Vision Science Group Graduate School of Health University of Technology Sydney Sydney NSW 2008 Australia; ^3^ Australian Institute for Microbiology and Infection University of Technology Sydney Sydney NSW 2008 Australia

**Keywords:** applications, bibliometrics, composition, magnetite, nanoparticles, reviews

## Abstract

Recent reports show air pollutant magnetite nanoparticles (MNPs) in the brains of people with Alzheimer's disease (AD). Considering various field applications of MNPs because of developments in nanotechnology, the aim of this study is to identify major trends and data gaps in research on magnetite to allow for relevant environmental and health risk assessment. Herein, a bibliometric and systematic analysis of the published magnetite literature (*n* = 31 567) between 1990 to 2020 is completed. Following appraisal, publications (*n* = 244) are grouped into four time periods with the main research theme identified for each as 1990–1997 “oxides,” 1998–2005 “ferric oxide,” 2006–2013 “pathology,” and 2014–2020 “animal model.” Magnetite formation and catalytic activity dominate the first two time periods, with the last two focusing on the exploitation of nanoparticle engineering. Japan and China have the highest number of citations for articles published. Longitudinal analysis indicates that magnetite research for the past 30 years shifted from environmental and industrial applications, to biomedical and its potential toxic effects. Therefore, whilst this study presents the research profile of different countries, the development in research on MNPs, it also reveals that further studies on the effects of MNPs on human health is much needed.

## Graphical abstract

Bibliometric and systematic analysis shows research from 1990 to 2020 reveals the versatility of magnetite nanoparticles and their evolving usage in a number of fields. Initially focusing on the environmental and industrial applications, to the biomedical applications. However, recent research highlights their toxicity and potential detrimental effects on human health.

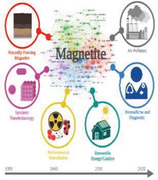

## 1 Introduction

Magnetite is an iron oxide (Fe_3_O_4_) rock mineral occurring naturally on Earth. Magnetite belongs to the spinel crystallizing group of minerals, with the formula FeFe_2_O_4_.^[^ ^1^ ^]^ In the natural environment, magnetite is found in the form of igneous (basic rocks, basalts) and sedimentary rocks (banded iron formations, beach sands) formed via different ways, usually, the reaction between ferric iron mats and ferrous iron anaerobically.^[^ ^2^ ^]^ Magnetite can readily react with oxygen to produce hematite (Fe_2_O_3_) where it can be used to determine oxygen concentrations in rocks, due to the changes in the atmospheric oxygen content.^[^ ^3^ ^]^ Magnetite exhibits ferrimagnetism where dipole forces align in the same direction, permitting active magnetization to occur. This property of magnetite is influenced by size of the particles and is essential for the reconstruction of tectonics plates on the Earth's crust.^[^ ^3^, ^4^ ^]^ Overall, magnetite is a unique iron oxide compound that is it extremely versatile due to its magnetic, structural, and redox characteristics.^[^ ^5^ ^]^

Material and life scientists have shown particular interest in substances at nano‐scale, referred to as nanomaterials or nanoparticles.^[^ ^6^ ^]^ Iron oxide magnetic nanoparticles or magnetite nanoparticles (MNPs) have become the focus for studies across various fields, including industrial, environmental, and biomedical applications of the nanoparticles. MNPs have been used extensively due to the fundamental properties of MNPs in the closely packed cubic lattice structure with the iron ions located at interstices between the oxygen ions, in either the tetrahedral or octahedral sites. The crystal structure of magnetite allows the movement from one ion to another (transitioning of valence states), exhibiting high conductivity, high catalytic performance, and regenerative abilities exhibiting ferromagnetic or superparamagnetic properties, which means that the external magnetic field can magnetize the particles to a paramagnetic (weakly attracted to magnets) but with much larger magnetic susceptibility.^[^ ^4^ ^]^ MNPs can be chemically synthesized and is relatively cheap to manufacture on a large scale, thus it is abundantly available.^[^ ^7^ ^]^ The pliability of synthetic MNPs has allowed them to be used in various environmental, industrial, and biomedical applications. For example, MNPs have been previously used to remove chromium, zinc, lead, arsenic, palladium, copper, and chloroform from polluted water and soil.^[^ ^8^, ^9^, ^10^, ^11^, ^12^ ^]^ In industries, MNPs (combined with carbon) have been shown to improve sodium and lithium battery life, improve rechargeable device efficiency, aid in the advancements of solar cells and biofuels due to thermal and catalytic properties.^[^ ^7^, ^13^ ^]^ In biomedical applications, superparamagnetic synthetic MNPs coated with organic materials has been shown to increase its stability and biocompatibility making it a safe and efficient biomedicine.^[^ ^6^, ^14^ ^]^ For example, in magnetic resonance imaging (MRI), MNPs have been used as a contrasting agent for tumor diagnosis, and hypothermia‐based cancer therapies and for inflammatory diseases.^[^ ^15^, ^16^, ^17^, ^18^, ^19^, ^20^ ^]^ The various applications of synthetic MNPs show their versatility, with manufacturer's exploiting the remarkable properties that they possess, which have been improved in many applications through the advancements in nanoparticle engineering and manufacturing.^[^ ^21^, ^22^ ^]^

Whilst magnetite research over the last 30 years has yielded progress in our understanding of the properties of magnetite, and the ways in which we can exploit its properties for various uses, there are aspects of MNPs which are unknown, which include the way in which they are harmful to human health.^[^ ^23^ ^]^ For example, a study by Maher et al. has shown that air pollutant‐externally‐derived MNPs have been found in abundance in the brains of people suffering from Alzheimer's disease (AD).^[^ ^24^ ^]^ The aim of this study therefore is to conduct a bibliometric analysis of the literature over the past three decades on MNPs related research and highlight future research needs.

## 2 Experimental Section

### 2.1 Topic Modelling

The database, Pubmed (https://pubmed.ncbi.nlm.nih.gov/), was used to search the term “magnetite” (938 publications). The publications that were within the years 1990–2020 and either a research or review articles were exported (PMID list file). They were then imported into the Sciome Workbench for Interactive Computer‐Facilitated Text‐mining (SWIFT) review software (https://www.sciome.com/swift-review/) where the articles were segregated based on keywords, then organized into topic models of magnetite research, and ranked in order from most to least prevalent topics. To enlarge the search range, other databases were used as a source of bibliographic data.

### 2.2 Bibliometric Analysis

The bibliographic data collected from a “magnetite” search including title, abstract, and all citations (accessed on 14th December 2020) for the period 1990–2020, was exported from the Web of Science Core Collection (WoS) database (txt. file). Duplicates identified were removed before all data were imported into the VOSviewer software (www.vosviewer.com). A bibliographic analysis was performed based on co‐occurrence of authors keywords in the paper title (11 949 keywords), using full counting, with a minimum of 20 occurrences in the dataset specified (134 keywords).

### 2.3 Longitudinal Study

**Figure 1:**
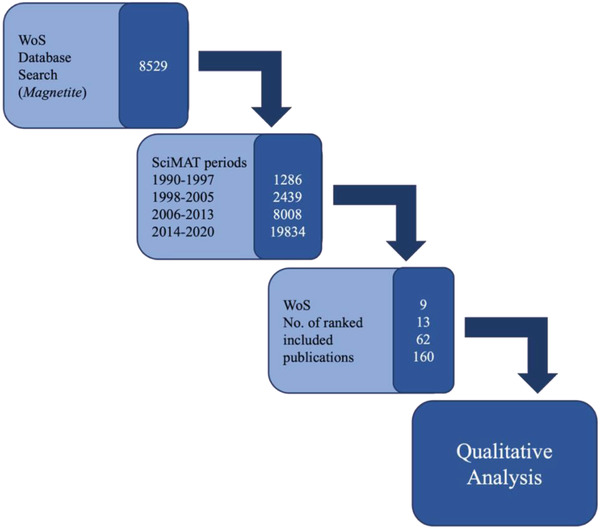
Graphical depiction of the systematic and bibliometric literature review process of the Web of Science (WoS) database (numbers represent the number of papers analyzed at that step).

To undertake a review of the literature for the research on “magnetite” over the past three decades, the bibliometric data present in Scopus (which included authors key words) for the topic “magnetite” (accessed on 14th December 2020) was obtained. This data (31 567 publications) was imported into Science Mapping Analysis Software Tool (SciMAT) (https://sci2s.ugr.es/scimat/) for analyses; singular and plural version of the same words were grouped. The publications were arbitrarily assigned to four time periods (1990–1997, 1998–2005, 2006–2013, 2014–2021) so that changes over this chronological period could be analyzed, using the workflow described (**Figure** **1**). Normalization was performed using the analysis function focusing on words and specifically authors keywords, with a frequency reduction minimum of 10, a co‐occurrence matrix, the edge value reduction of 8, normalization of association strength, simple algorithm centers, and core mapper, with quality measuring h‐index and the longitudinal analysis with an evolution map (using Jaccard's Index) and an overlapping map (using Inclusion Index). Important motor‐themes were identified by their location in the upper‐right hand quadrant of the strategic diagram generated by SciMAT software.

### 2.4 Systematic Review of Literature

A systematic review of the total available English language peer‐reviewed literature on magnetite in a Scopus database (date accessed on 14th December 2020) was carried out on the four main themes (e.g., magnetite AND oxides, 1990–1997) identified in SciMAT. Publications were restricted to the relevant time period related to the motor theme. The publications found were assessed according to the field citation ratio (FCR) for all time periods. A total of 244 publications were included in this analysis. A value for the FCR of greater than 6 (as a proxy indicating peer‐acknowledged quality) was used to justify inclusion of a publication in this study. Reviews, editorials, and short communication were excluded from further analysis.

### 2.5 Author Network and Citation Bursts

Co‐authorship networks were constructed in VOSviewer software using the same dataset from the WoS database (new format, RIS file), from the search on “magnetite” (accessed on 14th December 2020). The data was imported into the VOSviewer software, where a bibliographic database search was completed, for co‐authorship using full counting method, finding a total of 46 800 authors. The authors were further refined by a minimum of 14 documents and 20 citations per authors (used to as inclusion criteria for peer‐acknowledged quality, h‐index) resulting in 80 authors meeting this threshold. The authors affiliations and number of citations, along with the affiliated countries were recorded, and the percentage of citations per country were calculated to determine the frequency of “magnetite” research around the world. The percentage of citations per country was calculated and ordered by most to least.

## 3 Results

The term “magnetite” was searched in three different databases for scientific literature. A large difference in the number of publications was returned between each database, as seen in **Table** **1**. However, the trend for all searches was similar, with the term “magnetite” producing the highest number of publications for all databases. Scopus database returned the highest number (31 567 publications) of publications for all search terms compared to both WoS (8529 publications) and PubMed (938 publications). With the search term of “magnetite AND pathology” returning the smallest number of publications within the Scopus (2121 publications) and WoS (20 publications) database.

**Table 1:** Summary of the number of papers identified in searches of different databases in the years 1990–2020. Databases Web of Science (WoS), PubMed, and Scopus were accessed on 14th December 2020 and covered the article, title, abstract, and keywords.

Search terms	PubMed	WoS	Scopus
Magnetite	938	8529	31 567
(Magnetite) AND nanoparticle	741	1034	19 007
(Magnetite) AND pollution	22	58	4132
(Magnetite) AND pathology	222	20	2121

### 3.1 Topic Modelling

**Figure 2:**
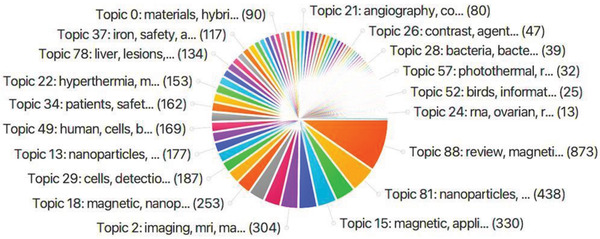
Topic models generated from PubMed dataset (938 publications) by SWIFT review software, using the search term “magnetite.” This search was refined to clinical trials, meta‐analysis, review, and systematic review articles. Accessed on 14th December 2020.

The dataset extracted on the PubMed database, from the search term “magnetite” was imported into the SWIFT review software for topic modelling. The articles were triaged based on keywords, categorized into topic models, and organized in ranking order. SWIFT review returned 100 topic models with an overview of the top 19 presented in **Figure** **2**. **Table** **2** presents the top 19 topic models and includes a summary description of each topic formulated to provide a theme for each model identified (based on the topic model in the review by Reichel et al. 2020).^[^ ^21^ ^]^ Most of the topics identified are associated with the magnetic properties and applications of MNPs with the latter mostly focusing on advanced imaging techniques. This search was completed to identify the main research themes of MNPs studies conducted from 1990 to 2020, and to identify the main focus and applications of their use.

**Table 2:** Top 19 topic models generated from PubMed dataset (938 publications) by SWIFT review software, using the search term “magnetite.” This search was refined to clinical trials, meta‐analysis, review, and systematic review articles. The topics have been ordered by number of publications contributing to the topic model in descending order, with topic words and themes established. Accessed on 14th December 2020

Topic number	Topic words	Number of publications contributing to topic model	Theme of topic model
88	Magnetic, applications, nanoparticles, review, recent, properties, biomedical, advances	873	Biomedical advances
81	Nanoparticles, clinical, applications diagnostics, therapeutics	438	Clinical applications
35	Magnetic, MNPs, biomedical, synthesis, properties, surface, imaging	340	Magnetic imaging properties
15	Iron, oxide, ultrasmall, MRI, superparamagnetic, contrast, imaging	330	Superparamagnetic property as contrast agent
27	Future, research, review, current, function, perspectives	319	Current research
2	Imaging, magnetic, resonance, function, probes, vivo, sensitivity	304	MRI use as contrast agent
23	Iron, oxide, nanoparticles, SPIONs, properties, applications, surface	285	Surface structure and properties
90	Size, magnetite, properties, synthesis, control distribution, specific, range	275	Formation and synthesis
18	Field, separation, particles, fields, external, application, surface, area, magnetic, nanoparticles	253	Conducting properties
3	Drug delivery, targeting, release, targeted, systems, anticancer, nanocarriers	217	Targeted drug delivery
29	Cells, detection, cancer targeting, early, specific, advanced, molecules, tumor, approach	187	Targeted cancer therapy
67	Studies, higher, data, larger, medical, form obtained, initial	178	Research analysis of magnetite
13	Clinical, agent, delivery, magnetic, therapy, gene, potential	177	Gene therapy agent
93	Agents, contrast, imaging, clinical media, tissue, extracellular	172	Clinical use to improve methodology
49	Human, cells, body, in vitro, external, material, found, toxicity, effect, tissues	169	Toxicity of MNPs to human cells
63	Nanoparticles, biological, inorganic, chemical, surface biomolecules, metal additives	169	Surface‐functionalized nanoparticles
34	Patients, safety, clinical, adverse, efficacy, event, injection, phase, safe, received	162	Clinical trials for biomedical applications
87	Dose, time, injection, effects, subjects, healthy, volunteers	154	Clinical trials for biomedical applications
22	Hypothermia, magnetic, treatment, cancer, therapy, heat	153	Hyperthermia‐based cancer therapy

### 3.2 Bibliographic Analysis

**Figure 3:**
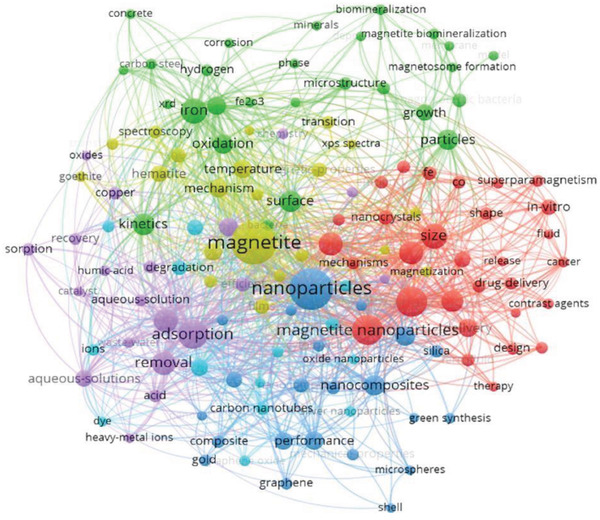
Network visualization map showing six color‐coded clusters produced using VOSviewer and the Web of Science (WoS) dataset, with the search “magnetite.” The network map shows analysis of 8529 publications from 1990 to 2020. The colors represent the clusters of keywords displayed referred to in Table 3. Accessed on 14th December 2020.

To examine the growth of literature in research associated with magnetite, the WoS database was used with the search term “magnetite” in the years 1990–2020, yielding 8529 publications. To create a visualization of the co‐occurrence of all keyword terms, the extracted dataset (title, abstract, and authors keywords) was imported into VOSviewer Software (Universiteit Leiden, Leiden, Netherlands, Version 1.6.15). The main characteristics obtained from an analysis of the co‐occurrence of keywords included the frequency and proximity of similar words. The keywords were refined by a minimum of 20 occurrences, resulting in 134 keywords which were organized by VOSviewer into six main clusters, seen in **Table** **3**. The main clusters have been organized to provide an overview of the main research that was carried out on “magnetite” from 1990 to 2020. Key discoveries within each cluster were identified with a lay description, seen in Table 3 and in a network visualization map, segregated by colors in **Figure** **3**. Cluster 1 (red) is focused on the biomedical applications of MNPs as a drug delivery system in cancer therapies and nanomedicine. Two of the clusters are closely linked to the magnetite formation and methodology used for research purposes (cluster 2 and 4; green and yellow respectively). Cluster 3 (blue) focuses on the antibacterial activity, morphology, and composition of MNPs whilst cluster 5 (purple) highlights the chemical adsorbent properties of magnetite in terms of environmental remediation. Cluster 6 (aqua) reveals MNPs additives (e.g., nanocomposites, core–shell, doped, and surface functionalized) for biomedical purposes. This analysis identified nanoparticle engineering enhancements and specific fields using nanotechnology (e.g., as biomedical therapies) which are further investigated.

**Table 3:** Summary of word clusters identified using VOSviewer and the WoS dataset obtained using a search for the term “magnetite.” The network analysis from 8529 publications from 1990 to 2020. The clusters are represented in a visualization map (refer to Figure 3). Accessed on 14th December 2020

Cluster	Lay/Description	Keywords
1 (red)	Cancer‐based therapies	Cancer, contrast agents, design, drug‐delivery, efficiency, ferrofluid, functionalization, hyperthermia, in‐vitro, magnetite nanoparticles, MRI, release, shape, size, superparamagnetic, therapy, toxicity
2 (green)	Formation, structure, and properties	Behavior, biomineralization, carbon steel, concrete, corrosion, deposition, Fe2O3, hydrogen, kinetics, magnetotactic bacteria, magnetosome formation, microstructure, minerals, Mossbauer spectroscopy, oxidation, phase, surface
3 (blue)	Surface and morphological alterations	Antibacterial activity, chitosan, cytotoxicity, decomposition, gold, graphene, green synthesis, mechanical‐properties, microspheres, nanocomposite, nanoparticle, performance, shell, silica
4 (yellow)	Synthesis, magnetic, and physical characteristics. Visualization methods	Conductivity, films, goethite, hematite, hydrothermal synthesis, maghemite, magnetic properties, magnetization, mechanism, Mossbauer, nanostructures, oxide, spectroscopy, system, temperature, transformation, transition, XPS spectra
5 (purple)	Environmental remediator properties and targets	Acid, adsorption, aqueous‐solution, catalyst, copper, efficient, heavy‐metal ions, humic acid, nanomaterials, nanotechnology, oxides, pH recovery, removal, stability, wastewater
6 (aqua)	Nanoparticle additives, and property characteristics	Carbon, nanotubes, dye, enhancement, graphene oxide, impact, interspecies electron‐transfer, ions, methane production, reduction, separation, silver nanoparticles

### 3.3 SciMAT

**Figure 4:**
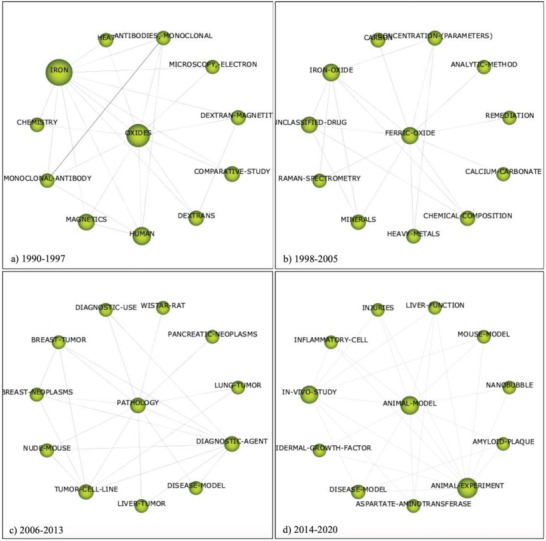
Main themes that emerged in magnetite‐related publications extracted from the Scopus database (31 567 publications), over four time periods using SciMAT: a) 1990–1997 (1286 publications); b) 1998–2005 (2439 publications); c) 2006–2013 (8008 publications), and d) 2014–2021 (19 834 publications). The figure shows the links between the keywords within the four major themes identified for each time‐period. Accessed on 14th December 2020.

Scientific research on magnetite has changed significantly over the last three decades, with studies initially investigating the thermal and oxidative properties of MNPs, then shifting focus onto the biomedical applications as a drug delivery and diagnostic agent, eventually looking into the effects of MNPs on the central nervous system, as seen in **Figure** **4**. This shift is reflected in the longitudinal study performed using the SciMAT software with the Scopus database (31 567 publications). Between 1990 and 1997 (1286 publications) the term “oxides” is associated with terms like “magnetics,” “dextran,” and “heat,” indicating an additive of dextran coating of MNPs, with electron microscopy appearing as a common technology for sedimentary formation analysis of MNPs.^[^ ^21^ ^]^ In the period of 1998–2005 (2439 publications) the term “ferric oxide” emerged as the main theme that is associated with words such as “remediation,” “unclassified drug,” and “carbon.” In this period, Raman spectroscopy was a common technology used for soil analysis and chemical composition for remediation purposes. Of note in this period, studies were beginning to investigate the potential of MNPs as experimental drug delivery agents.

During 2006–2013 (8008 publications) the term “pathology” associated with “diagnostic use” emerged as the major theme. In line with this theme, high‐frequency keywords including “tumor” and “diagnostic‐agent” were present, with various animal models mentioned (“Wistar rats” and “nude mice”) along with tumor cell lines, being used for experimental studies. In 2014–2021 (19 834 publications), analysis showed “animal model” as the main theme in association with terms like “inflammatory cell,” “liver function,” and “amyloid‐plaque” for studies of this period, suggesting that research focused on the role of MNPs in inflammatory and neurodegenerative diseases. The trajectory of MNPs research, initially focuses on the formation and characteristics of doped MNPs, as an anti‐cancer agent, eventually leading to the in vivo and in vitro experiments highlighting the potential toxicity and role in neurodegenerative diseases.

### 3.4 Systematic Review of Literature

**Figure 5:**
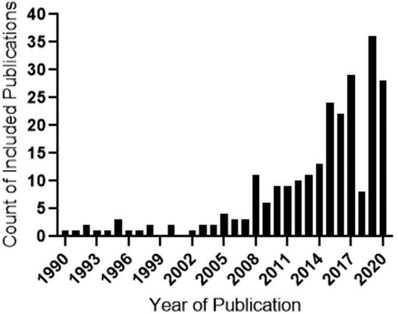
Frequency histogram of 244 publications derived from a systematic search of magnetite literature, conducted in the Scopus database for the search term “magnetite” AND oxides/ferric oxide/pathology/animal model, showing a peak publication between 2015 and 2020. The year 2019 demonstrated the highest number of publications. Accessed on 14th December 2020.

The systematic literature analysis, on the Scopus database was completed to provide an analysis of literature from each theme established in the SciMAT search. This yielded a total of 244 scientific publications, refined by a citation ratio of greater than 6 (peer‐acknowledged quality threshold). The first time period (1990–1997) produced 9 publications (“oxides”), the second (1998–2005) produced 13 publications (“ferric oxide”), the third time period (2006–2013) produced 62 publications (“pathology”), and the last time period (2014–2020) produced the most with 160 publications (“animal model”), seen in **Figure** **5**.

#### 3.4.1 Time Period 1990–1997 (Magnetite and Oxides)

This period yielded nine publications extracted from the literature search with four publications that focused on magnetite formation in sediment and its ability to leach chemical pollutants from soil and groundwater^[^ ^25^, ^26^, ^27^, ^28^ ^]^ One publication studied magnetite as an oxidizing agent to better understand the reactivity of crystalline iron minerals in marine sediment formation.^[^ ^26^ ^]^ Two publications focused on magnetite isotope characteristics in sediment for geothermometers to measure temperature in deep sea deposits.^[^ ^27^, ^28^ ^]^ Three publications focused on the biomedical application of MNPs coated with polypeptide, polyoxymethylene‐polypropylene copolymers, ferumoxides, ferumoxtran, or ferumoxsil (dextran coated, dextran covered, or siloxane coated, respectively) found to be superior for MRI contrasting and cell labelling compared to bare superparamagnetic MNPs (SPIONs).^[^ ^29^, ^30^, ^31^ ^]^ Last, two publications highlighted the chemical properties and mechanisms of magnetite transitioning between valence states.^[^ ^26^, ^32^ ^]^ The range of research on magnetite in this period covered many fields with the majority focused on naturally forming magnetite and the mechanistic properties that magnetite particle possess. This period also highlighted the use of coated MNPs as a biomedicine, which are all continued in the successive time periods.

#### 3.4.2 Time Period 1998–2005 (Magnetite and Ferric Oxide)

A total of 13 publications were included in this time period, with some overlapping themes to the previous period; MNPs for environmental remediation and as a contrasting agent for MRI.^[^ ^33^ ^]^ Five publications highlighted formation, regenerative abilities, and abundance of magnetite in soil, with two studies investigating whether there is a link between the decrease in anerobic respiration, and another study focusing on magnetic properties in soil relating to future environmental change.^[^ ^34^, ^35^, ^36^, ^37^, ^38^ ^]^ Three publications highlighted environmental remediation studies using of MNPs with hydroxide coatings to extract arsenic and other heavy metals from soil, assessing the effects of particle size, surface texture, and morphology.^[^ ^39^, ^40^, ^41^ ^]^ Three publications studied the valence electron transfer mechanism in MNPs coated with various organic compounds and its potential application to have increased regenerative abilities and improved catalytic capacity to increase battery charge.^[^ ^42^, ^43^, ^44^ ^]^ Two publications focused on MNPs and its potential as a biomedicine with one study adding bipolar surfactants to the high temperature synthesis reaction resulting in transition from hydrophobic to hydrophilic nanoparticles, increasing dispersibility for biomagnetic applications (e.g., MRI).^[^ ^45^ ^]^ The second study used surface‐functionalized MNPs with hydrophilic organic molecules (e.g., tetramethylammonium hydroxide [N(CH_3_)_4_OH]) and reported enhanced MRI contrasting effects, increase cell viability in in vitro models (monkey kidney Cos‐7 cells) and whole human blood samples. The surface modified/functionalized MNPs led to increased dispersion of the nanoparticles, enhancing contrasting abilities.^[^ ^33^ ^]^ While most of the publications in this period focused on the characterization and physicochemical biological effects of MNPs in soil and as an environmental remediator, the advancements in the design and engineering of the nanoparticles for biomedical purposes were also significant, the latter being the main theme of research in the next period.

#### 3.4.3 Time Period 2006–2013 (Magnetite and Pathology)

A total of 62 publications were included in this time period, with overlapping themes from the previous period where MNPs were investigated in removing contaminated waste in soil, as MRI contrasting agents and improvements battery life. The studies on the use of MNPs for environmental remediation increased substantially in this period (19 publications) targeting the removal of methylene blue, copper, chromium, arsenic, and chlorophenol as waste contaminants from water and soil.^[^ ^46^, ^47^, ^48^, ^49^, ^50^, ^51^ ^]^ These MNPs were coated with various materials (e.g., methylene blue) or as nanocomposites (various compounds incorporated into a matrix of standard materials; ceria/MNPs, graphene oxide/MNPs, and reduced graphene oxide/MNPs) and investigated the nanosheet structure for enhanced catalytic activity to degrade contaminants in soil.^[^ ^11^, ^47^, ^48^, ^50^, ^52^, ^53^ ^]^

Three publications referred to MNPs and their role as a biosensor in a variety of biomedical, environmental, and industrial applications. One study utilized MNPs as a colorimetric biosensor, and proved to be extremely sensitive, detecting allergies, toxins in water, and chronic diseases.^[^ ^54^ ^]^ The second, highlighted graphene oxide/MNPs nanocomposites, found to have improved dispersibility, compared to bare MNPs, thereby increasing electrocatalytic properties, indicating promise for energy.^[^ ^55^ ^]^ Last, a study used dopamine‐coated MNPs, being found to improve electrochemical efficiency and in turn, increasing lithium battery life.^[^ ^56^ ^]^

Most of the publications in this period described the biomedical applications of MNPs (31 publications). Many studies (16 publications) focused on improving the design and manufacturing of MNPs so they are more effective in vivo.^[^ ^52^ ^]^ This was achieved by increasing the surface‐to‐volume ratio of MNPs and specifically SPIONs, which alter the magnetic property (e.g., as the size decrease, the magnetic anisotropy energy decreases; anisotropy is the energy that keeps the magnetic particles in a certain orientation) of MNPs.^[^ ^57^, ^58^ ^]^ Studies investigating the morphology of MNPs demonstrated that more compact nano‐cubes have less oxygen vacancies and are more stable, compared to more oxygen vacancies, less stable, and less compact nanorods and nanowires.^[^ ^52^, ^59^, ^60^ ^]^ There is limited evidence to suggest superior morphology, however the most widely used is nano‐cubes and nanospheres, with one study suggested that nanowires were extremely effective hyperthermia‐based cancer therapy.^[^ ^57^, ^58^ ^]^ The composition of MNPs is the most commonly cited parameter, which is dependent on the synthesis method (e.g., gas phase, liquid phase, microemulsion, sol‐gel, facile and solvothermal, and thermal decomposition), nature of the dopant (e.g., magnetic or non‐magnetic), which can affect the stability, dispersibility, coercivity (resistance of a material to changes in magnetization), and functionality of the nanoparticles.^[^ ^52^, ^61^, ^62^ ^]^ The composition is highly specific for the purpose of the nanoparticles (e.g., core–shell design also impacts the characteristics of MNPs, enhancing biocompatibility) providing another avenue for maximizing coercivity, and therefore biocompatibility.^[^ ^58^, ^63^ ^]^ Other biomedical applications of MNPs are more specialized, with one publication focusing on surface‐functionalized MNPs with protamine sulfate, which gives a cationic surface charge on the magnetic particles, enhancing the transfection efficiency in an in vitro (mouse pLG2 liver cells) and in vivo (BALB/c mice, liver) model based on HVJ‐E technology, finding that surface modifications can enhance gene transfer (via magnetic targeting) and potentially overcome gene therapy. Two publications investigated SPIONs, dextran coated (found to increase nanoparticle presence in blood circulation), mesoporous silica, (for controlled drug delivery, isolation of genomic and plasmid DNA), amorphous silica (isolation of biomolecules, drug delivery), and polyethylene glycol^[^ ^64^ ^]^ as a nanowire (drug delivery agent, targeting drug resistant cancer cells).^[^ ^60^, ^63^, ^65^, ^66^ ^]^ Coating the MNPs with polymer‐like materials (e.g., dextran and PEG) increases the targeting efficiency of SPIONs, showing these coating materials as a potential transport carrier across the blood–brain barrier (BBB).^[^ ^65^ ^]^ Three publications were found to focus on hyperthermia‐based therapies and drug delivery applications, with one publication highlighting core–shell MNPs (used for chemotherapy, radiation, and immunotherapy) and magnetic microcapsules (used for hyperthermia and drug delivery), finding that the latter was highly specific and also remained in circulation for a longer time.^[^ ^67^ ^]^ Similarly, another publication focuses on the potential for multi‐functional modalities of hyperthermia and drug delivery having magnetite as the nanoparticle core with a polymer shell, as a nanocomposite, with targeting agents on the surface. This MNP design allowed for more controlled drug release, increased loading and greater stability of MNPs.^[^ ^68^ ^]^ Another publication found that nano‐cubic iron oxide particles designed specifically for hyperthermia‐based therapy demonstrated superior magnetic heating efficiency as the cubic form particles were able to retain heat more readily than other particle shapes of similar size.^[^ ^69^ ^]^

One publication reviewed the macrophage recognition to evade the immune response, however also identified the cytotoxic effects of MNPs in vivo (nude mice), finding that oxidative stress was increased, regardless of the composition and even mild exposures of MNPs. The observed toxicity was found to be size‐dependent, and if small enough would escape phagocytosis, and lie within macrophages, promoting oxidative stress and reactive oxygen species (ROS) mediated activator protein 1(AP‐1) and nuclear factor kappa B (NFκB) activation.^[^ ^70^ ^]^ This indicates the gaps in knowledge of MNPs as a biomedicine, which is further explored in the following period.

The research on MNPs in this period, provided insight into the physical changes of MNPs, and the physicochemical characteristics of the nanoparticles for specific environmental, biomedical, and industrial application. The various advances in doping, coating and adjusting the size, morphology, and composition in this period was extensive, however the use of nanocomposites prevailed as the most common and effective nanostructure in this period. Combining these manipulations is seen in the later years of this period, which is followed through in the next.

#### 3.4.4 Time Period 2014–2020 (Magnetite and Animal Model): Environmental, Industrial, and Biomedical Applications

This period yielded the highest number of publications (160 publications) making up more than 50% of all included publications in the final literature appraisal. The period also saw the highest number of publications (36 publications) from a single year (2019). The broad themes for this period are again on the environmental and industrial applications, with significant focus on the biomedical applications as well as toxicological studies of air pollutant MNPs. Many studies (nine publications) in this period described the synthesis process, characterization, and engineering of MNPs in nanocomposites.

Publications focusing on the environmental applications of MNPs was the most extensive (76 publications). Research (24 publications) highlighted the ability of graphene oxide, cerium oxide, graphene carbon, silver, silica, carbon, and polymer like/MNPs nanocomposites ability to efficiently adsorb heavy metals (e.g., chromium, lead, copper, caladium, mercury, arsenic, uranium, and nickel), aromatic compounds (e.g., phenazopyridine, tetracycline, and sulfamethazine) and toxic dyes (e.g., methylene blue, organic dyes from polluted waste water, and sediment).^[^ ^71^, ^72^, ^73^, ^74^, ^75^, ^76^, ^77^, ^78^, ^79^, ^80^ ^]^ Microwave adsorption using nanocomposites was also extensively studied (seven publications) with graphene, carbon, yolk–shell, and molybdenum disulfide/MNPs nanocomposites.^[^ ^81^, ^82^, ^83^ ^]^ All these nanocomposites have proven to be highly efficient due to the added activated oxygen sites exposed on the surface of these nanocomposites which readily form stable chelates with heavy metal ions and adsorb microwaves.^[^ ^72^, ^81^, ^84^ ^]^ Other publications (five publications) focused on MNPs with a core–shell of poly(m‐phenylenediamine), manganese, and graphene oxide to adsorb/remove pollutants (chromium, cationic dyes, lead, copper, and arsenic) from wastewater.^[^ ^71^, ^76^, ^85^, ^86^ ^]^ These publications investigated different types of MNPs configuration including nanospheres, nanofibers, nanosheets, nanotubes, nanoflowers, and nanoring, and textured surface for their distinct capacities to remove pollutant moieties.^[^ ^81^, ^82^, ^84^, ^87^, ^88^, ^89^, ^90^ ^]^ The nanosphere structure has been most widely used for this purpose, however nanosheets, nanoflower‐like and nanoring structures are highly efficient adsorbers as more surface area increases active sites. Similarly, the more porous a surface the more adsorptive.^[^ ^81^, ^82^, ^88^, ^90^, ^91^, ^92^ ^]^

Many of the advances used for environmental applications of MNPs are also used for industrial applications focusing on renewable energy, with one publication using the catalytic nature of MNPs to increase methanogenic propionate degradation for renewable energy production.^[^ ^93^ ^]^ Nanocomposites are also widely used in this field with graphene oxide, sodium potassium, and graphene aerogel combined with MNPs for enhanced electrochemical conductivity and supercapacitor performance (for efficient energy storage and usage).^[^ ^94^, ^95^, ^96^ ^]^ Other studies used MNPs nanocomposites with lithium–sulfur (enhanced electrical conductivity, improved battery life in lithium ion batteries) and polymethyl methacrylate‐ functionalized MNPs (found to be flame retardant and be efficient electromagnetic wave adsorbents) as the structure enabled greater dispersion, and recyclable ability.^[^ ^94^ ^]^

The biomedical applications of MNPs in this period are extensive, with subtopics reappearing in this period like contrasting agents, applications for therapy in cancer and drug delivery agents. MNPs as a contrasting agent in this period, focused on SPIONs, with one study using a small SPION agents (coated with carbohydrates), ferumoxide, which can be taken up by macrophages and the reticuloendothelial system to image lymph nodes and certain tumors for MRI imaging up to 11 months after injection.^[^ ^97^ ^]^ Other studies have tested core–shell SPIONS coated with amino‐functionalized octahedral carboxylate shells, further functionalized with PEG. This modification increased sensitivity, low toxicity, good biocompatibility, and degradability both in vitro (KB cells – human epithelial carcinoma cells) and in vivo (BALB/C mice bearing KB tumors) making it a promising MRI contrasting agent.^[^ ^21^, ^98^ ^]^ Last, hyaluronic acid‐modified MNPs/Gold (core/shell) nano‐stars were investigated in in vitro (U87MG cells) and in vivo (BALB/c nude mice) models as an imaging and photothermal therapy of tumors, finding that the nanoparticles are water dispersible, colloidally stable, and biocompatible and therefore have great potential as a contrasting agent.^[^ ^99^ ^]^

The use of MNPs as cancer treatments and drug delivery agents are extensively investigated in this period. Many studies (ten publications) highlighted the use of MNPs in various forms (magnetite fluid, hyaluronic acid‐modified magnetite gold core/shell, coated with PEG, as ferumoxytol and SPIONs), which accumulate in and around the tumor effectively killing the cancer cells.^[^ ^21^, ^99^, ^100^, ^101^, ^102^, ^103^ ^]^ The photothermal nature (Fenton reaction) of MNPs initiate the production of ROS and the application of heat which work to kill cancer cells have been seen in in vitro (A549 cells and MMTV‐PrMT‐derived mammary carcinoma cells) and in vivo (female FVB/N mice) models as well as early mammary cancers and cancer metastases in the liver and lungs.^[^ ^16^, ^104^, ^105^, ^106^ ^]^ These studies found that the biocompatibility and physiological stability was improved, along with faster clearance enhanced permeability.^[^ ^101^, ^107^, ^108^ ^]^ Other publications used mesoporous silica (further functionalized with PEG) and gold, polymer‐like shell loaded with doxorubicin that enhanced MNPs photothermal efficiency in killing cancer cells.^[^ ^5^, ^109^, ^110^ ^]^ Using MNPs as a diagnostic testing tool have been studied with immobilized surface antigen Hepatitis B on the surface of carbon‐coated MNPs for better detection of Hepatitis B in serum and protein for diagnostic use.^[^ ^111^ ^]^

Other biomedical applications of note in this period focus on the immune response, bone regeneration, and diagnostic testing methods. One publication uses MNPs’ surface functionalized with oleic acid to form a complex that is able to diagnose egg allergies.^[^ ^112^ ^]^ Another study uses dextran‐coated SPIONs core–shell nano‐worms incubated in human serum, to aid the in removal of proteins which may trigger an immune response to allergies, offering sight into safer nanomedicines for human subjects.^[^ ^113^ ^]^ MNPs have also been used for bone regeneration studies, using porous poly‐lactide/polyglycolic acid (PLLA/PGA)/MNPs nanocomposite to stimulate bone regeneration, tested on in vitro (MG63 cells) and in vivo (New Zealand white rabbits) models, finding that the bone tissue formation was significantly accelerated, along with increase cell proliferation and differentiation.^[^ ^114^ ^]^ Of particular interest is the synthesis of molecularly imprinted magnetite nanozymes, with one publication finding that it can improve specificity, activity, and mimic peroxidase‐like activity as a drug delivery method, and another study using a nanozyme MNPs/carbon core–shell nanocomposite, in the form a nanowires, for an assay to enhance signal amplification, and detection of platelet‐derived growth factor, in human serum samples in order to replace more costly.^[^ ^115^, ^116^ ^]^ Another study used PEG‐coated MNPs, for the detection of hydrochlorothiazide (anti‐hypertensive agent) from human urine as a low cost, water compatible, and environmentally friendly method.^[^ ^117^ ^]^ It is apparent that advances in nanoparticle engineering and their exploitation of the physicochemical characteristics in conjunction with many other compounds for customized biological functions, has driven the significant progress of MNPs biomedical research in this period.

The advancements of nanoparticle engineering in this period are clear, with the extensive use of surface‐functionalized, coated, nanocomposite or a combination of these structural components. The biomedical applications of MNPs in this period are of particular interest, showing improvements in drug delivery, and cancer therapies, but also incorporating MNPs in a wide range of diagnostic testing methods, along with aiding and evading the immune response (e.g., allergies and antimicrobial resistance).

#### 3.4.5 Time Period 2014–2020 (Magnetite and Animal Model): Toxicity

In relevance to the in vivo studies, toxicity publications (two publications) also surfaced in this period, describing the potential impact of MNPs use on human health. As the extensive use of MNPs in various fields is established, the studies show that nanoparticles including MNPs, may be toxic in many fields that use them. With publications finding the potential genotoxic (toxic to DNA) effects of MNPs with various coatings; polyaspartic acid (bone marrow cells), silica oxide (Hep3B cells), dextran (MCL5 human lymphoblastoid cell line), and bare MNPs (L‐929 murine fibroblasts).^[^ ^118^, ^119^, ^120^, ^121^, ^122^ ^]^ Of concern is bioaccumulation, biodistribution, and toxicity of various nanoparticles showing damage to cell membranes by increasing permeability, inducing cytotoxicity, and in the environment, inhibiting photosynthesis.^[^ ^12^, ^123^ ^]^ Scholarly research has also investigated the long‐term repercussions of MNPs, those originating from anthropogenic sources, in neurodegenerative diseases. Ultrafine air pollutant MNPs have been previously implicated in cardiovascular diseases and neurodegenerative diseases (i.e., AD), as particles smaller than ≈200 nm can enter the heart via the circulatory system, and the brain via the olfactory nerve through the BBB.^[^ ^124^, ^125^ ^]^ One publication detected presence of MNPs in the brains of AD patients, deducing that the nanoparticles were inhaled air pollutants with distinct spherical morphology and smooth surface texture unlike those of biological origins (from iron metabolisms) with octahedral morphology.^[^ ^21^ ^]^ Excess magnetite in the brain causes toxicity, increasing oxidative stress, and ROS, found to be near amyloid‐beta (Aβ) fibrils in the AD brain.^[^ ^24^, ^125^, ^126^, ^127^, ^128^ ^]^ The toxicity studies highlighted the extensive use of MNPs in a variety of fields and their impact on human health, which is consistent with the recent evidence that air pollutant MNPs have been implicated in various diseases, including neurodegeneration (i.e. AD).

### 3.5 Author Network and Countries with Most Citations For “Magnetite” Research

Over the past 30 years, the direction of research into magnetite has been influenced by the catalytic and kinetic properties of magnetite, which has enabled the extensive application in the environmental, industrial, and biomedical fields, due to the advancements in technologies to direct the research through its manipulation for specific applications across various fields. To evaluate the authors involved in the advancements of magnetite research, an author network was established using VOSviewer software with the dataset obtained from the search “magnetite” in the years 1990–2020 from the WoS database (8529 publications). A bibliographic analysis for co‐authorship (full counting method) refining authors with a minimum of 14 documents and 20 citations per author (used to as inclusion criteria for peer‐acknowledged quality, h‐index) resulted in 80 authors meeting this threshold.^[^ ^129^ ^]^ Figure S1, Supporting Information, represents these authors in an overlay visualization network providing the years in which these authors have published. The author's affiliations and number of citations were recorded and the percentage of citations per country was calculated and ordered by most to least citations (**Table** **4**).^[^ ^130^ ^]^ The top 13 countries were established, showing the highest number of citations from Japan (22%), followed closely by China (21%), suggesting that these countries have led the world in magnetite research over the past 30 years. Other countries like Germany (13%), USA (11%), Iran (6%), France (5%), Romania (4%), Brazil (3%), Singapore, United Kingdom (2%), Spain, Hungary, and Republic of Korea (1%) have also contributed to magnetite research over the past 30 years.

**Table 4:** Top 13 countries with the highest percentage of citations (minimum 20) and publications (minimum 14) of authors, grouped by country affiliations and in descending order. Extrapolated from the dataset obtained from the Web of Science (WoS) database using the term “magnetite” in the years 1990–2020. Accessed on 14th December, 2020

Rank	Country of affiliation	Number of citations	% Citations
1	Japan	8331	22%
2	China	7826	21%
3	Germany	4704	13%
4	USA	4064	11%
5	Iran	2279	6%
6	France	1685	5%
7	Romania	1337	4%
8	Brazil	1091	3%
9	Singapore	821	2%
10	UK	752	2%
11	Hungry	530	1%
12	Korea	492	1%
13	Spain	472	1%

## 4 Discussion

**Figure 6:**
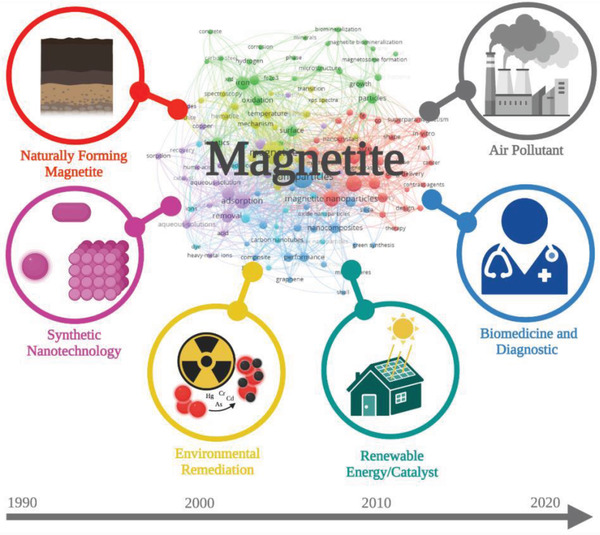
Timeline showing research on magnetite nanoparticle shifting from 1990 to 2020.

This systematic literature analysis revealed that scholarly studies on “magnetite” have been increasing worldwide with the highest percentage of publications originating from Japan and China. The results show that research on “magnetite” have shifted over the last 30 years (**Figure** **6**), from initially focusing on the formation of naturally occurring magnetite, into the synthetic development and manipulation of nanoparticles for various environmental, industrial, and biomedical applications. Ultimately, to the advancements in biomedical applications which uncover the potential toxicity of MNPs and air pollutant MNPs being implicated in various diseases including neurodegeneration and AD.^[^ ^24^, ^126^, ^131^ ^]^ Progress in MNP engineering has allowed applications in environmental remediation, batteries and solar cell, diagnostic and cancer therapy applications through tuning of the nanoparticles size, shape, and surface components, as well as, equally important, the development of MNPs core–shell, nanocomposite, and hybrid nanostructures.

The VOSviewer software was used to analyze literature and identify clusters, highlighting magnetite research which focused on the kinetic and catalytic properties of MNPs, the adsorptive abilities, and remediation targets as an environmental tool and the biomedical applications of MNPs (largely cancer‐based therapies highlighting the surface and morphological alterations). The literature analysis using SciMAT software established themes for each time period; the first time period from 1990 to 1997 identified magnetite AND oxides, with environmental remediation and biomedical applications of MNPs as the main themes. The environmental studies in this period mainly described magnetite distribution in soil, its crystalline reactivity structure and its ability to adsorb contaminants (e.g., arsenic).^[^ ^25^, ^26^, ^27^ ^]^ The biomedical applications of MNPs in this period are most interesting, with most publications referring to doped/coated MNPs. Doping is a widely used method for modifying the nanoparticles to enhance their electrical, optical, and biological activities.^[^ ^132^, ^133^ ^]^ Doping provides more stability to the nanoparticle structure, as the dopant increases the hydrophilicity of the nanoparticle, therefore increasing dispersibility and in turn reducing cytotoxicity, thus improving biocompatibility.^[^ ^134^ ^]^ There are various synthesis methods, and parameters (e.g., temperature, dipping time, and nature of surfactant) that affect the coating thickness, and therefore the overall size of the nanomaterial. For example, increasing concentration of silica (i.e., thicker coating) allows greater dispersion of nanoparticles and enhanced MRI contrasting performance with the use of an external magnetic field.^[^ ^65^ ^]^ Organic compounds, including surfactants and polymers represent a good choice for coating MNPs. In particular, coating with dextran, a complex branched polysaccharide polymer chain unit of various lengths (from 1 000 000 000 Da), has surfaced as advantageous with its biodegradable and water‐soluble properties further enhancing MNP biocompatibility.^[^ ^135^ ^]^ Coating with dextran 1) prevented nanoparticle agglomeration and toxicity of magnetic particles and 2) increased dispersibility and rapid clearing by macrophages from the blood, liver, spleen, and lymph nodes, compared to bare MNPs.^[^ ^29^, ^136^ ^]^ Dextran is unique as it allows magnetite's magnetic structure to be extremely strong at the surface where strong magnetic disorder is usually occurs.^[^ ^135^ ^]^ Doping/coating of MNPs is observed to be extremely useful finding that there is reduced cytotoxicity, and consequently improving the potential application for diagnostic and therapeutic purposes, which is further investigated in later time periods.^[^ ^137^ ^]^

The studies in the second period (1998–2005) focus primarily on the environmental and biomedical applications of MNPs, particularly focusing on the engineered surface chemistry. Both fields further explore the effects of coated MNPs, with adjustments to the surface (surface functionalization/modification) and morphology of the nanostructures. The environmental application of hydroxide‐coated MNPs indicated that the cubic spinel structure of MNPs (a cubic lattice structure, whereby Fe (III) ions occupy the tetrahedral and octahedral sites) renders the nanoparticles more hydrophilic and therefore, dispersing relatively easily in aqueous biological systems, enhancing the adsorptive properties. This is consistent with a hydrophilic polymer (polyethylene oxide)‐coated MNP study, in which the adsorption of heavy metals was improved due to the increased dispersion rate in solution.^[^ ^138^ ^]^ The biomedical applications exploring surface modification/functionalization in this period is extensive, specifically referring to the surface chemistry (i.e., increasing, decreasing, or neutralizing the surface charge) which strongly influences the properties of the system (e.g., polymers, small molecules, surfactants, dendrimers, and biomolecules).^[^ ^139^, ^140^, ^141^ ^]^ This occurs through the treatment of nanoparticles with specific agents, and is extremely valuable in enhancing cellular utilization, cellular uptake, decreased toxicity, improved binding capacity, enabling selectivity and specificity, with longer retention time, or non‐interactions (for evading the immune response) depending on its specific function.^[^ ^139^, ^140^ ^]^ Polymer surface‐functionalized NPs are extremely versatile, with the hydrophilic coating allowing increased circulation time. One study used dextran‐coated cross‐linked (altering the charge to be more positive) surface‐modified MNPs, which reduced drug concentrations at non‐target sites, due to increased dispersibility, overall resulting in fewer drug side effects.^[^ ^141^ ^]^ Another study used surface modification of MNPs with antibiotics, revealing that they could bind to bacterial cell walls, altering the cell wall integrity, due to its positive charge, demonstrating effective antimicrobial abilities. This was found to improve the antimicrobial inhibition against the pathogenic bacteria *Escherichia coli, Staphylococcus aureus*, and *Pseudomonas aeruginosa*.^[^ ^140^, ^142^ ^]^ Like doping or coated additives, surface modification/functionalized, also increases biocompatibility by altering the surface charge, and the nanoparticles can disperse more easily, and evade the immune response, due to the biocompatible additives on the surface, increasing reactive sites and for biomolecule attachments. This is further examined in later time periods, targeting the immune system, transportation of modulating agents, and as potential vaccines.^[^ ^143^ ^]^

In the 2006–2013 period, studies on MNPs focused on various morphological additives, particularly the use of nanocomposites due to the small size, increased dispersibility, enhanced adsorption, and solar energy function for environmental and industrial applications.^[^ ^54^ ^]^ Nanocomposites are nanoparticles incorporated into a matrix, displaying improvements in thermal stability, increased surface defects, chemical resistance, and improved electrical conductivity.^[^ ^47^ ^]^ For example, graphene oxide/MNP and polymers/MNP nanocomposites were found to have increased adsorptive capabilities of heavy metals and aromatic compounds, due to the increased surface area (increased surface defects) of this matrix structure.^[^ ^79^, ^81^, ^144^ ^]^ The biomedical applications of MNPs, was the major research theme in this period, with MNP nanocomposites also being considerably investigated. The use of nanocomposites have been found to be superior to doped/coated nanoparticles, as they behave more similarly to superparamagnetic nanomaterials, with a study displaying that polymer‐like (PLGA)/MNP nanocomposites are more effective tumor targeting MRI contrasting agents compared to dextran‐coated MNPs of similar size.^[^ ^145^ ^]^ Another study using mesoporous dye‐doped silica MNP nanocomposites, loaded with doxorubicin (potent anti‐cancer drug), found an improvement in MRI contrasting and tumor targeting abilities, whilst also being able to induce targeted cancer cell death.^[^ ^63^ ^]^ The combination of adding various nanoparticles into a nanocomposite structure, offers unique properties that are suggested to arise from the interactions of these materials in the matrix. This further proves the advancements in nanotechnology engineering with improved thermal stability, dispersibility, and increased surface exposure, thereby improving compatibility as a biomedicine, which is further explored in the successive time‐period, targeting the immune system, transportation of modulating agents, but also as potential vaccines.^[^ ^143^ ^]^

The most recent 2014–2020 period showed a shift in MNPs research with less proportion of publications dedicated on environmental remediation, and a clear surge in industrial and biomedical applications. MNP nanocomposite structures are again explored in this period, however there is a trend in studies focusing on the advancement in combining two or more additives (e.g., coating/doping, core–shell, and surface modification/functionalized) into nanocomposite matrix, termed “hybrid.” The nanocomposite hybrid was investigated for industrial applications with one study, using lithium–sulfur‐coated MNP nanocomposites, surface functionalized with carbon, finding that the catalytic activity excelled due to the increased surface defects, enhancing recovery rate, thereby increasing battery life.^[^ ^146^ ^]^ In addition to focusing on the nanocomposite structure, the biomedical applications of MNPs in this time period, also focus on the MNP hybrid nanocomposites as more efficient contrasting agents for MRI and drug delivery agents for cancer therapies (e.g., hyaluronic acid‐modified gold/MNPs nanocomposites) but extending further into the biomedical field by advancing the engineering of nano systems to overcome immunological barriers.^[^ ^99^ ^]^ The immune system responds to nanoparticles triggered through mechanisms that recognize surface molecules, peptides, or foreign materials, modulating an immune response based on the size, morphology, surface characteristics, and charge of the nanostructure. Therefore, evading the immune response relies on several properties of nanotechnological engineering, for example, reducing nanostructure size, adjusting morphology to a more biocompatible shape (i.e., nanosphere compared to nanocubes), and adding surface additives that increase the circulation time and overall biocompatibility.^[^ ^147^ ^]^ The manufacturing additives that can be applied to MNPs nanocomposites amplify the “stealth” ability, increasing circulation time, by which point they can provide active and specific targeting in the body.^[^ ^148^ ^]^ One study found that poly(caprolactone)‐coated MNPs loaded with doxorubicin hydrochloride, further surface functionalized with Arg–Gly–Asp enzymes, behaved like SPION nanocomposite microspheres, enhancing circulation time, whilst also exhibiting a quick magnetic response, advantageous for controlled drug release at tumor target site.^[^ ^149^ ^]^ This further confirms MNP nanocomposite hybrids as promising cancer and immune‐modulatory therapies. The development of nanotechnology in the past three decades, has discovered the study of nanoparticle additives and most recently nanocomposite hybrids appear to fulfil the growing needs of multifunctional materials, being extremely versatile, with the most increased dispersion and retention rate, as well as the binding force and wear of the nanostructure.^[^ ^150^ ^]^

Toward the final years of the 2014–2020 period, several research inquiries reported potential toxic effects of MNPs, being implicated with the environmental and biomedical applications. For example, the use of MNPs for environmental remediation used in wastewater and sediment treatment has led to extensive run off leading to an accumulation in aquatic environments. This accumulation leads to ROS/reactive nitric species (RNS) production, causing damage to exposed organisms, stimulating delays of hatching, damage in cell wall and outer membranes, and depletion of oxygen exchange and hypoxia to an in vitro model (rainbow trout spermatozoon).^[^ ^151^ ^]^ For biomedical applications, the cytotoxic effect of MNPs were tested using in vivo and in vitro models showing activation of AP‐1 and NFκB pathways that subsequently results in increased oxidative stress and induced apoptosis regardless of dosage or composition.^[^ ^70^, ^152^ ^]^ MNPs coated with oleic acid and silica, were found to exhibit cytotoxicity in in vitro models (human neuroblastoma SH‐SY5Y and glioblastoma A172 cells) through a decrease in cell viability.^[^ ^153^ ^]^ Similarly, MNPs coated with silica and polymer‐like materials for the purpose of cell labelling and hyperthermia treatments were also found to permeate cells, accumulating and causing cellular dysfunction.^[^ ^154^ ^]^ While these studies show the disadvantages of MNPs, the cytotoxic effects were shown to be dose dependent, with one study finding that MNPs cause toxicity to an in vitro model A549 cell line only at concentrations of greater than 50 µg mL^−1^ and that silica‐coated MNPs caused cellular dysfunction by impairing cellular adhesion properties at 0.1 µg µL^−1^.^[^ ^152^, ^154^ ^]^ Therefore, establishing a safe and effective dose of MNPs as a biomedicine should be more closely investigated to prevent adverse side effects.

Toxicity studies also focused on the biological effects of air pollutant MNPs, more specifically on their potential links with the progression of neurodegenerative diseases like Parkinson's disease and AD.^[^ ^3^, ^155^ ^]^ Biogenic magnetite can be found in a wide range of organisms from bacteria to animals, including humans.^[^ ^156^ ^]^ Physiologically biogenic magnetite has been found to aid in the biological metabolism of iron.^[^ ^20^, ^157^ ^]^ Interestingly, Maher and colleagues showed that anthropogenic (synthetic) MNPs can be found in abundance in the brains of people suffering from AD, distinguished by differences in their morphology to biogenic MNPs.^[^ ^21^ ^]^ MNPs, like other air pollutant particulate matter, is formed through frictional pressure at high temperatures. It is therefore no surprise that MNPs can be found in abundance in automotive diesel exhaust, grinding of trains on railway lines, welding factories, petroleum exhaust, and coal emission.^[^ ^158^ ^]^ Due to the formation process of these air pollutant MNP (<200 nm), they can migrate into the central nervous system via the nasal canal and olfactory bulb.^[^ ^155^ ^]^ Inhaled air pollutant nanoparticles in the brain tissues are thought to trigger ROS/RNS production, leading to oxidative stress.^[^ ^159^, ^160^, ^161^ ^]^ This increase in oxidative stress, is believed to further contribute to chronic inflammation, and consequently increase Aβ fibril formation found near Aβ plaques in AD, however, this is not confirmed. Therefore, consequent future studies should focus on understanding the effects of air pollutant MNPs in neurodegenerative diseases including AD.

## 5 Conclusion

In summary, the scholarly research on magnetite over the last three decades has generated knowledge of how the characteristics of MNPs affect the nanoparticle physical, catalytic, and biological activities, and in turn, how the knowledge is being used to guide the engineering of the nanoparticles for specific environmental, industrial, and biological applications.^[^ ^106^ ^]^ For the latter, being the most explored applications of MNPs, the tuning of the nanoparticle characteristics is also aimed to optimize biocompatibility, more specifically, for less aggregation, lower toxicity as well as enhancing cellular targeting and uptake, for use in disease diagnostic and therapy.^[^ ^162^ ^]^ Optimizing the engineering of NPs for biomedical applications has proven difficult, with the ability of the NPs to remain in circulation to achieve a desired goal, and on the other hand remaining too long in circulation causing toxicity, is a balance that has yet to be perfected. Future applications of MNPs in the biomedical field should focus on adjusting the size and morphology of the nanostructure, the “stealth” mechanism to evade the immune response which makes them an effective therapy or detection of human diseases. Considering the increasing evidence on the roles of air‐pollutant MNPs and the onset and progression of neurodegenerative diseases, the next phase of research should also focus on elucidating the exact mechanisms of the nanoparticle‐induced stress, including activation of inflammatory markers, while also focusing on epidemiology studies to determine any correlation between highly polluted sites and disease prevalence.

## Conflict of Interest

The authors declare no conflict of interest.

## Supporting information

Supporting Information

## Data Availability

The data that supports the findings of this study are available within the article itself.
