# Supplementary material for: Systematic and Bibliometric Analysis of Magnetite Nanoparticles and Their Applications in (Biomedical) Research
Source: Glob Chall. 2022 Sep 14;7(1):2200009. doi: 10.1002/gch2.202200009 (PMC9818080; doi:10.1002/gch2.202200009)
Supplement: Supplementary file 1 — Supporting Information [file GCH2-7-2200009-s001.pdf]

Global Challenges

Supporting Information

for *Global Challenges,* DOI: 10.1002/gch2.202200009

Systematic and Bibliometric Analysis of Magnetite Nanoparticles and Their Applications in (Biomedical) Research

Charlotte L. Fleming, Mojtaba Golzan, Cindy Gunawan, and Kristine C. McGrath**

Supporting Information

Systematic and Bibliometric Analysis of Magnetite Nanoparticles and Their Applications in (Biomedical) Research

Charlotte L. Fleming[1], Mojtaba Golzan[2], Cindy Gunawan[3], Kristine C. McGrath[1*]*

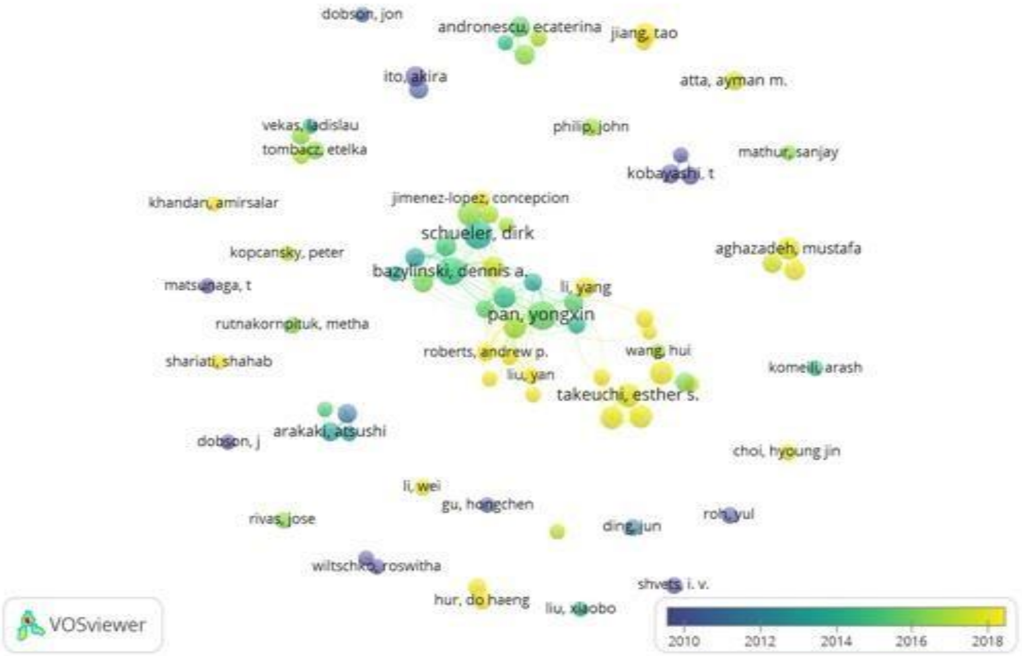

Figure S1. Overlay visualisation map of the author network obtained from the Web of Science (WoS) dataset using the VOSviewer software, with the search term “magnetite”. The network map shows the top 80 authors meeting the publication minimum of 14 and citation minimum of 20 from 1990-2020. The colour scale represents the year which these authors published (e.g. purple indicates 2010). Accessed on 14th of December 2020.
